# Distribution of estrogenic steroids in municipal wastewater treatment plants in Tehran, Iran

**DOI:** 10.1186/2052-336X-12-97

**Published:** 2014-06-20

**Authors:** Azita Mohagheghian, Ramin Nabizadeh, Alireza Mesdghinia, Noushin Rastkari, Amir Hossein Mahvi, Mahmood Alimohammadi, Masoud Yunesian, Reza Ahmadkhaniha, Shahrokh Nazmara

**Affiliations:** 1 Department of Environmental Health Engineering, School of Public Health, University of Medical Sciences, Tehran, Iran; 2 Center for Air Pollution Research (CAPR), Institute for Environmental Research (IER), Tehran University of Medical Sciences, Tehran, Iran; 3 Center for Water Quality Research (CWQR), Institute for Environmental Research (IER), Tehran University of Medical Sciences, Tehran, Iran; 4 Center for Solid Waste Research (CSWR), Institute for Environmental Research (IER), Tehran University of Medical Sciences, Tehran, Iran; 5 Department of Human Ecology, School of Public Health, Tehran University of Medical Sciences, Tehran, Iran

**Keywords:** Endocrine disrupting compounds, Solid-phase extraction, Estrogenic steroids, Municipal wastewater treatment plant

## Abstract

**Background:**

Estrogenic steroids such as estrone (E1), 17β–estradiol (E2), estriol (E3), and 17α–ethinylestradiol (EE2) are among the most potent endocrine disrupting compounds (EDCs). Compared with North America, Europe and Japan there is no reliable information on the concentration of steroid hormones in wastewater treatment plants (WWTPs) influents and effluents in Iran. The aim of the present study was to determine the amounts of E1, E2, E3, and EE2 influents and effluents of 7 municipal WWTPs across Tehran, the capital city of Iran, in two seasons, summer and autumn, through solid-phase extraction (SPE) gas chromatography–mass spectrometry (GC–MS).

**Results:**

The results showed that the concentrations of E1, E2, and EE2 in influents ranged from 6.54–18.76 ng/L, 1.02–8 ng/L and 4.18–11.76 ng/L, respectively. Also, the concentrations of E1, E2, and EE2 in effluents ranged from 1.04–4.99 ng/L, 0.5–2.20 ng/L and 0.5–2.58 ng/L, respectively. The levels of E3 were below the detection limit (0.5 ng/L). The percentage removal rate of E1, E2 and EE2 ranged between 61.76–87.25%, 50.98–82.63%, and 66.3–90.25%, respectively. Results indicated no significant correlation between hormone concentrations and seasons.

**Conclusions:**

The study showed that WWTP number 7 had significant differences in influent hormone concentrations compared with others. Results only showed a significant relationship between hormones and TSS removal rate, but there was no significant relationship between hormones and COD removal rate. The removal rate of hormone in WWTP number 4 and 7 were significantly different from the others. There was no significant correlation between hormone concentrations and seasons.

## Introduction

Endocrine disrupting compounds (EDCs) include several types of natural and synthetic chemicals that mimic or prevent the endocrine system in animals and human beings and disrupt the function of these glands [1]. EDCs mainly include synthetic and natural hormones and their metabolites, some personal care products and pharmaceuticals, several non-steroidal drugs, synthetic compounds that are used as flame retardants, plasticisers, and pesticides [2]. Among the different classes of endocrine disrupters, natural and synthetic estrogens, such as estrone (E1), 17 beta–estradiol (E2), estriol (E3), and 17α–ethinyl estradiol (EE2) have much stronger estrogenic effects than other EDCs [3].

The environmental concentrations of estrogens are very low; however, the presence of estrogenic compounds in the environment has become a concern, because they may interfere with the reproduction of human beings, livestock and wildlife. Concentrations below 0.1 ng/L of one estrogen are sufficient to cause significant estrogenic effects [4]. Estrogens have a stimulating effect on breast tumour growth [5] and about 95% of breast cancers are known to be hormone dependent [6]. They also cause endometrial cancer and ovarian and other hormone cancers [7].

Treated effluents from WWTPs are thought to be major pathway for these contaminants as treatment facilities are not designed to capture or remove such as diverse range of chemical pollutants [4]. Estrogenic steroid hormones were found in WWTPs at concentrations of 2.4–670 ng E1/L [8,9], 2.4–150 ng E2/L [10,11], <1.8–660 ng E3/L [9,12], and <0.3–70 ng EE2/L [9,11] in influents, and <0.3–96 ng E1/L [10,11], 0.2–30 ng E2/L [9,10], 0.44–275 ng E3/L [9,13], and <0.3–5 ng EE2/L in effluents [9,11].

Due to the health effects of estrogenic compounds, since municipal wastewater is the main disposal pathway for the human waste born estrogenic compounds and because of the lack of reliable information about this component at the time of this study in Iran, the present study was performed on the determination the amounts of E1, E2, E3, and EE2 in the raw sewage influent and final treated effluent of 7 municipal wastewater treatment plants across Tehran by solid-phase extraction (SPE) gas chromatography–mass spectrometry (GC–MS).

## Materials and methods

### Descriptions of the wastewater treatment plants (WWTPs)

Information about the operation conditions and conventional wastewater parameters of each WWTP are summarised in Table 1. Three types of secondary plants are used in these WWTPs: suspended growth-activated sludge with extend aeration, trickling filter followed by an activated sludge tank and an anaerobic/anoxic/oxic activated sludge process (A^2^O)which removes biological phosphorus with simultaneous nitrification – denitrification. Conventional wastewater parameters such as 5-day biochemical oxygen demand (BOD_5_), total chemical oxygen demand (COD), total organic carbon (TOC) and total suspended solids (TSS) were analysed according to the standard methods.

**Table 1:** Operation conditions of 7 municipal wastewater treatment plants (WWTPs)

**WWTP no.**	**1**		**2**		**3**		**4**		**5**		**6**		**7**	
**Biological treatment**	AS/EA^a^		AS/EA		AS/EA		AS/EA		CAS^b^		A^2^O AS^c^		TF/AS^d^	
**People served**	7,000		42,000		30,000		20,000		85,000		100,000		2,100,000	
**Mean influent flow (m**^**3**^**/h)**	50		200		200		100		1200		670		15000	
**Person (L/d)**	171.5		114.28		160		120		338.8		160.8		166	
**Total HRT (h)**	15		28		10		15		12		24		9	
**Aeration tank HRT (h)**	12		20		6		8		4		15		6	
**SRT (d)**	-		15-20		12		-		12-18		20-30		20	
**Sample period**	S^*e*^	A^*f*^	S	A	S	A	S	A	S	A	S	A	S	A
**Mean influent COD (mg/L)**	264	152	160	304	140	120	170	312	192	216	400	200	432	515
**Mean influent BOD (mg/L)**	200	130	90	130	90	90	155	285	160	185	155	125	230	235
**Mean influent TOC (mg/L)**	110	70	68	97	73	68	75	135	87	91	98	68	161	175
**Mean influent TSS(mg/L)**	180	160	100	230	50	315	120	515	145	325	250	225	145	230
**Mean effluent COD (mg/L)**	12.8	16	9.6	16	16	12.8	6.4	12.8	12.8	16	41.6	12.8	17	27
**Mean effluent BOD (mg/L)**	8	14	8	8	3.5	8	2.4	4.5	5.8	8	10	7.1	8.5	6.2
**Mean effluent TOC (mg/L)**	4.7	7	7.5	9	4.5	9.5	3.5	5.8	8.5	12.3	15.3	9.5	10	13.5
**Mean Effluent TSS(mg/L)**	22	20	10	26	15	10	1	8	17.5	30	11	17	5	8
**Influent temperature (°C)**	24.3	23.3	25.7	22.8	23.6	20.5	24.8	22.6	24.9	22.7	25.9	23.8	26	25.5
**Effluent temperature (°C)**	23.9	22.7	25.6	22.4	23.9	20.7	25	22.1	24.6	19.7	25.9	23.2	24.4	24

^a^Activated sludge/extended aeration, ^b^Conventional activated sludge, ^c^The A^2^O process is composed of an anaerobic/anoxic/oxic process which removes biological phosphorus with simultaneous nitrification-denitrification, ^d^Trickling filter followed by activated sludge, ^e^22, 25, 28 July 2012, ^f^22, 25, 28 October 2012.

### Sampling and preparation of samples

Grab samples were manually collected over 6 days (22, 25 and 28 July and 22, 25 and 28 October) from the raw sewage influent and final treated effluent of 7 municipal wastewater treatment plants in Tehran by using brown glass bottles with Teflon stoppers. The samples were transferred to laboratory by a cool box at 4°C. The sewage samples (1 L) were filtered through GF/F filters (0.7 and 0.2 μm) then spiked with etiocholanolone as an internal standard. Samples extraction was performed by using a solid phase extraction system according to the established procedures presented by Zhang et al. [14].

Methanol as a solvent was purchased from Merck (Darmstadt, Germany). The compounds E1, E2, E3, EE2, and etiocholanolone were purchased from Sigma (UK) and N,O–bis(trimethylsilyl)trifluoroacetamide (BSTFA) containing 1% of trimethylchlorosilane (TMCS), was supplied by Aldrich (Dorset, UK). Internal standard solutions (100 ng/L) of etiocholanolone were prepared in methanol. Ultrapure water was supplied from a Millipore Ultrapure water system.

The SPE C18 cartridges (1000 mg/12 mL–Teknokroma) were conditioned with 12 mL of methanol which was passed through the cartridges under a very low vacuum to ensure that the sorbents were soaked in methanol for 15 min to remove residual bonding agents. Then, ultrapure water was passed through the cartridges at a rate of 1–2 mL/min. Water samples were extracted at a flow rate less than 5 mL/min. The cartridges were dried under vacuum for 30 min and then the analytes were eluted into vials (20 mL) from the sorbents with 12 mL of methanol at a flow rate of 1 mL/min. The solvents were blown down to 1 mL under a gentle flow of nitrogen at less than 50°C. The extracts from SPE were transferred into 1.5 mL reaction vials. The extracts were further evaporated to dryness under a gentle nitrogen stream. The dry residues were derivatised by the addition of 80 μL of BSTFA and 20 μL of pyridine. After a reaction time of 30 min at 60–70°C, 10 μL of final extract was injected into GC/MS apparatus.

### GC-MS analysis

The instrument used for GC-MS analysis was a 3800 Varian gas chromatography coupled to a Varian Saturn 2200 mass spectrometer, equipped with an HP-5 capillary column (30 m, 0.25 mm i.d., 0.25 μm film thickness). The instrumental temperatures were as follows: injector temperature 280°C; transfer line 300°C; initial oven temperature 80°C (held for 0.5 min), increased to 250°C at a rate of 20°C min^−1^ to 300°C at a rate of 5°C min^−1^ and hold at 300°C for 4 min. The inlet was operated in split on mode. The temperature of the transfer line was maintained at 290°C.

Helium (99.999%) was used as a carrier gas at 1 mL min^−1^ (constant flow). The source and quadrupole temperatures were kept at 230 and 150ºC, respectively. The electronic beam energy of the mass spectrometer was set at 70 eV. The mass selective detector was operated in electron impact (EI) mode using selected ion monitoring (SIM). The dwell time of each ion was set at 100 ms. The GC conditions were selected to minimise the time of analysis while allowing all of the analytes to elute in acquisition groups containing a suitable number of ions for monitoring.

The detection limit for E1, E2, E3, and EE2 was 0.5 ng/L. All of the laboratory analyses on hormones were carried out in the Central and Chemistry Lab at the School of Public Health, Tehran University of Medical Sciences. Statistical analysis of the obtained results was performed according to linear regression, chi-square, Kruskal-Wallis, Tukey HSD and one-way ANOVA analysis.

## Results and discussion

In current research the amounts of E1, E2, E3, and EE2 in the raw sewage influent and final treated effluent of municipal wastewater treatment plants were determined. Table 2 illustrates the descriptive statistics summary of steroid hormones.

**Table 2:** Descriptive statistics summary of steroid hormones

**Function**	**E1 Influent**	**E2 Influent**	**EE2 Influent**	**E1 Removal**	**E2 Removal**	**EE2 Removal**
Mean	11.37	3.01	6.22	71.82	68.18	80.43
Standard deviation	3.03	1.70	1.94	5.41	6.78	4.49
Sample variance	9.21	2.89	3.77	29.27	45.95	20.14
Minimum	6.54	1.02	4.18	61.76	50.98	66.33
Maximum	18.76	8.00	11.76	87.25	82.63	90.25
Confidence level (95.0%)	0.95	0.53	0.61	1.69	2.11	1.40

As Table 2 illustrates the concentrations of E1, E2, and EE2 in influents varied from 6.54–18.76 ng/L, 1.02–8 ng/L, and 4.18–11.76 ng/L, respectively, with mean concentrations of 11.37 ± 3.03 ng/L, 3.01 ± 1.70 ng/L, and 6.22 ± 1.94 ng/L, respectively. The levels of E3 were below the detection limit (0.5 ng/L). In many researches, similar to the research presented here [15], E3 was not detected in influents. Other researchers [13,16-19] reported the levels of estrogens in WWTP influents to range from non-detectable (nd) to 66.0 ng E1/L, nd −22.7 ng E2/L, nd–80.0 ng E3/L, and nd–7.1 ng EE2/L. The concentrations of E1, E2, and EE2 in effluents varied from 1.04–4.99 ng/L, 0.5–2.20 ng/L, and 0.5–2.58 ng/L, respectively, with mean concentrations of 3.15 ± 0.81 ng/L, 0.91 ± 0.47 ng/L, and 1.21 ± 0.42 ng/L, respectively. The concentration of estrogens in WWTP effluents was reported from nd–82 ng E1/L, nd–64 ng E2/L, 0.4–39.1 ng E3/L, and nd–42 ng EE2/L in Sweden [20], Canada and Germany [21], Italy [13], the UK [17,22], The Netherlands [11], Japan [23], and China [24,25] . According to the results of this study, only EE2 concentrations in influent were higher than other values reported by other researchers. In other cases, hormone concentrations were less in both influent and effluent.

**Figure 1:**
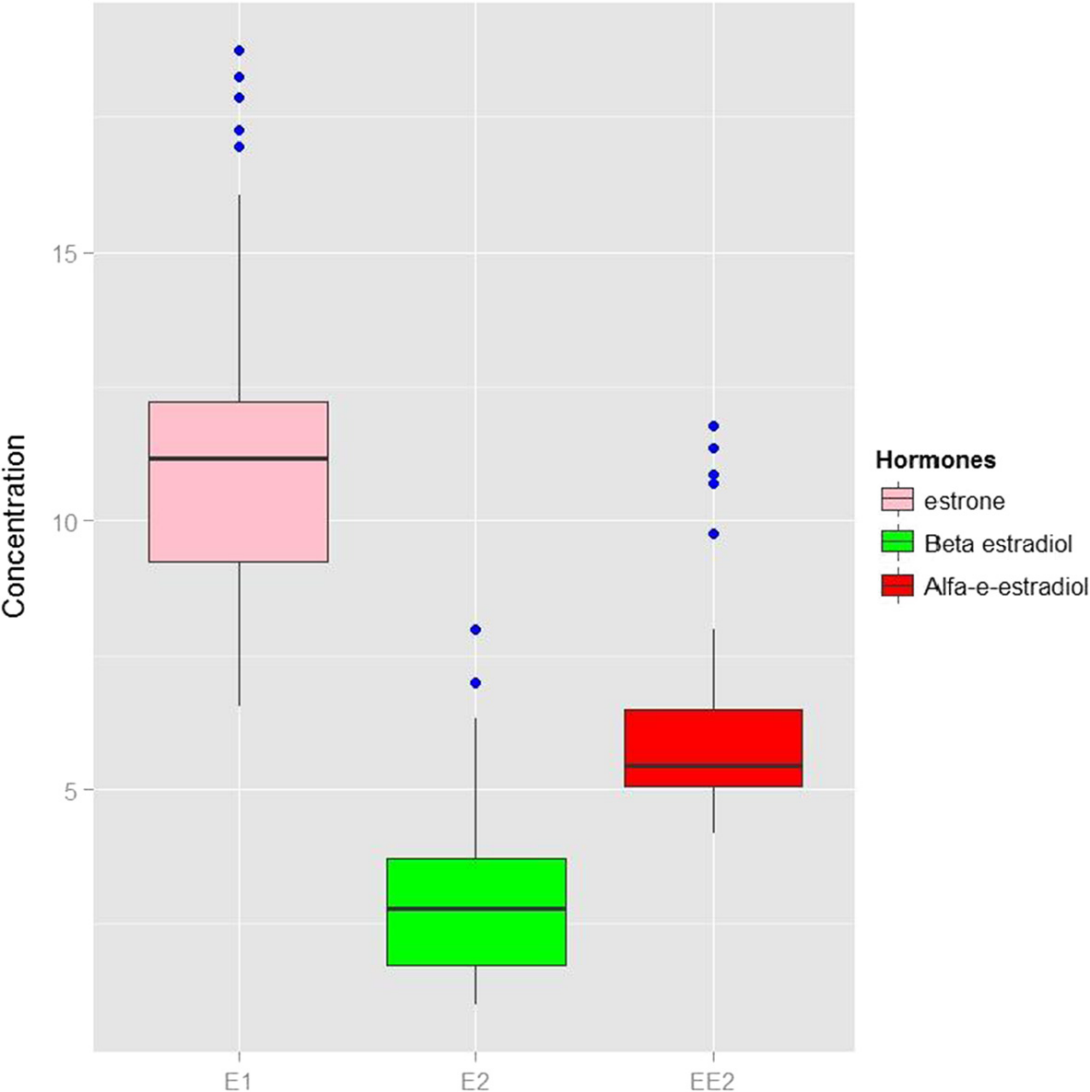
Influent hormone concentrations vs. types of hormone (mean concentrations of 11.37 ± 3.03 ng/L for E1, 3.01 ± 1.70 ng/L for E2 and 6.22 ± 1.94 ng/L for EE2).

In this study, hormone concentrations results were analysed by ANOVA test. Results showed that hormone concentrations in influents of these WWTPs significantly varied from case to case (p = 2.24 × 10^−5^). Figure 1 illustrates hormone concentrations versus types of hormone.

The concentration of different hormones, in different WWTPs, was analysed by Tukey HSD test (Table 3).

**Table 3:** The results of Tukey HSD test of hormone concentrations at wastewater treatment plants

**WWTP**	**Diff**	**Lwr**	**Upr**	**p adj**
1-2	0.076	−3.655	3.806	1.000
1-3	−1.328	−5.059	2.402	0.936
1-4	0.193	−3.538	3.924	1.000
1-5	0.461	−3.270	4.191	1.000
1-6	0.842	−2.889	4.573	0.994
1-7	5.382	1.651	9.112	0.001
2-3	−1.404	−5.135	2.327	0.918
2-4	0.117	−3.614	3.848	1.000
2-5	0.385	−3.346	4.116	1.000
2-6	0.767	−2.964	4.497	0.996
2-7	5.306	1.575	9.037	0.001
3-4	1.521	−2.210	5.252	0.884
3-5	1.789	−1.942	5.520	0.780
3-6	2.171	−1.560	5.901	0.587
3-7	6.710	2.979	10.441	0.000
4-5	0.268	−3.463	3.999	1.000
4-6	0.649	−3.081	4.380	0.998
4-7	5.189	1.458	8.920	0.001
5-6	0.382	−3.349	4.112	1.000
5-7	4.921	1.190	8.652	0.002
6-7	4.539	0.809	8.270	0.007

According to Table 3 only WWTP number 7 showed a significant difference in influent hormone concentrations compared with others (P_value_ < 0.001). WWTP number 7 serves a community with a population of 2,100,000. Its influent flow treatment rate is about15000 m^3^/h and it treats much more wastewater than the other WWTPs.

The higher steroid estrogen concentrations in the WWTP influent and effluent may be due to the differences in treatment plant catchment characteristics, including commerce-industry–domestic sewerage mix, treatment technology used, higher population density, higher birth rate, less dilution, different sampling times and other socioeconomic factors [17,26].

In this research, the percentage removal rate of E1, E2, and EE2 ranged between 61.76–87.25%, 50.98–82.63%, and 66.3–90.25%, respectively, with mean concentrations of 71.82 ± 5.41 ng/L, 68.18 ± 6.78 ng/L, and 80.43 ± 4.49 ng/L, respectively. In various WWTP, the removal rates for estrogens were reported to be 23–83% for E1, 59–100% for E2, 80–99% for E3 and 71–78% for EE2 [27]. It is almost similar to present study.

It is necessary to note that the concentration and removal percentage of estrogenic steroid obtained in different studies are not easily comparable; because the conditions of wastewater treatment plants are different and/or sometimes not clearly described. In addition, sampling strategies and analytical methods from one study to another are different [17].

The relationship between COD and TSS removal rate with hormone removal rate was examined by linear regression. Results showed only a significant relationship between hormone removal rates and TSS removal rates (R–Squared = 0.99; p = 2.2 × 10^−16^). There was no significant relationship between hormone removal rate and COD removal rate (p = 0.585).

The relationship between hormone removal percentage and type of WWTP were examined by ANOVA test. The results showed that the relationship between some of them is significantly different (p = 0.001). The percentage removal rate of hormones in different WWTPs was analysed by Tukey HSD test. Table 4, shows the results of the Tukey HSD test. As shown in Table 4, the hormone removal rate in WWTP number 4 is significantly different from WWTP number 1 and 3. The hormone removal rate of WWTP number 7 was also significantly different from WWTP number 3 compared with other WWTPs.

**Table 4:** The results of Tukey HSD test on hormone removal at wastewater treatment plants

**WWTP**	**Diff**	**Lwr**	**Upr**	**P adj**
1-2	0.33	−6.80	7.45	1.00
1-3	−2.50	−9.62	4.63	0.94
1-4	7.36	0.23	14.48	0.04
1-5	2.70	−4.42	9.82	0.92
1-6	2.40	−4.72	9.53	0.95
1-7	5.20	−1.93	12.32	0.31
2-3	−2.82	−9.95	4.30	0.90
2-4	7.03	−0.10	14.15	0.00
2-5	2.37	−4.75	9.50	0.95
2-6	2.08	−5.05	9.20	0.98
2-7	4.87	−2.25	11.99	0.39
3-4	9.85	2.73	16.98	0.00
3-5	5.20	−1.93	12.32	0.31
3-6	4.90	−2.22	12.02	0.38
3-7	7.69	0.57	14.82	0.43
4-5	−4.66	−11.78	2.47	0.04
4-6	−4.95	−12.08	2.17	0.03
4-7	−2.16	−9.28	4.96	0.04
5-6	−0.30	−7.42	6.83	1.00
5-7	2.50	−4.63	9.62	0.94
6-7	2.79	−4.33	9.92	0.90

As mentioned before, this study showed a significant relationship between hormone removal rates and TSS removal rates. WWTP number 4 and WWTP number 7 had the highest removal rate for TSS compared with the others (98.81 and 96.54%, respectively). Therefore, significant differences in the hormone removal rate in WWTP number 4 and WWTP number 7 may be due to their high rate of TSS removal rate. It should be mentioned that the wastewater treatment process in WWTP number 7 was trickling filter followed by activated sludge which has a long sludge retention time.

Johnson and Sumpter [17] reported sorption on an organic-rich solid phase and biodegradation are major mechanisms of estrogenic steroids removing in biological treatment.

As Auriol et al. [27] reported, the long sludge retention time has a positive effect on activated sludge system for removing estrogens.

Secondary treatment with activated sludge with longer sludge and hydraulic retention times has a very good estrogenic steroid removing rate, up to 90% [28].

**Figure 2:**
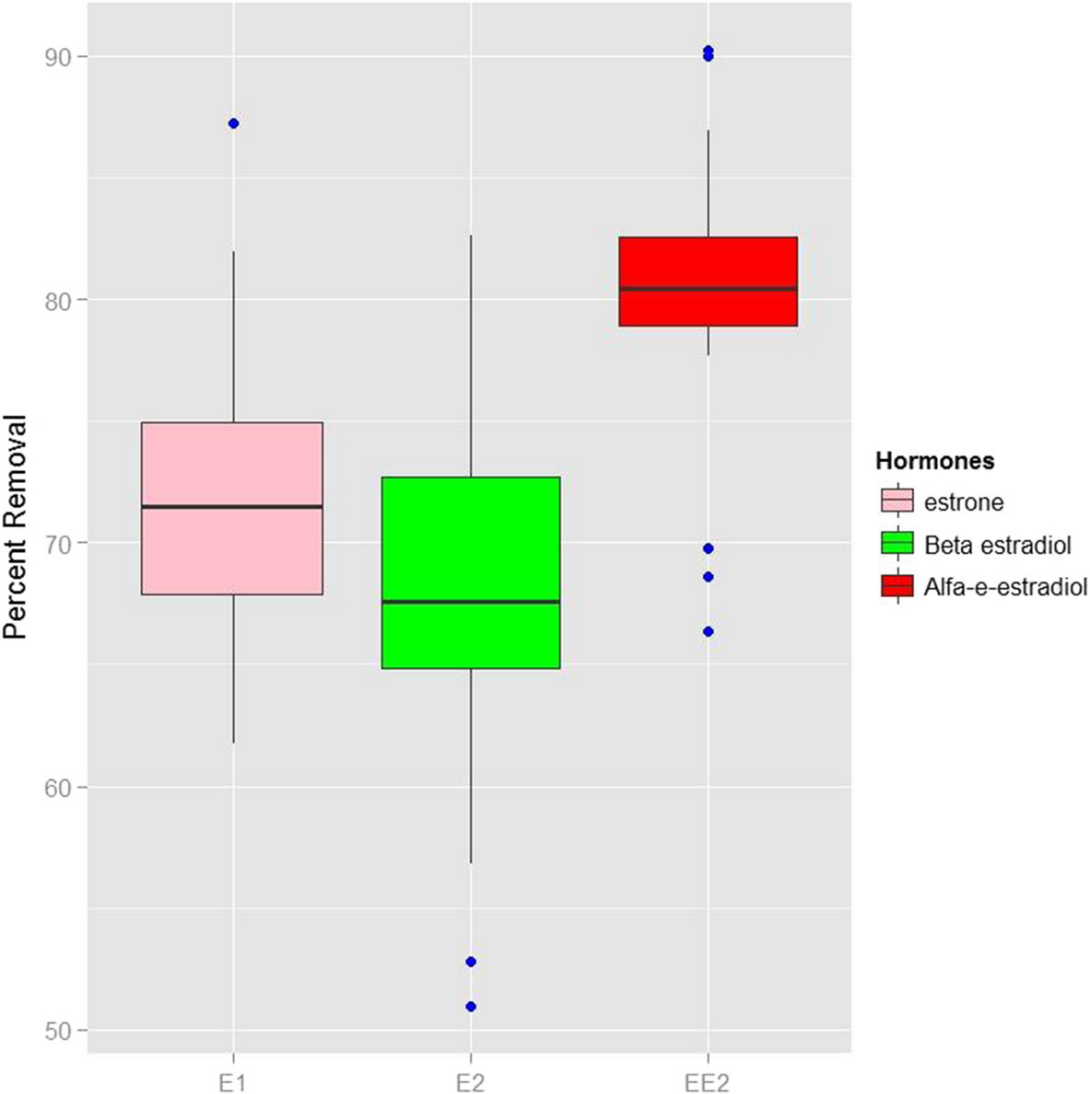
Removal percentages of hormones vs. types of hormone (mean concentrations of 71.82 ± 5.41 ng/L for E1, 68.18 ± 6.78 ng/L for E2 and 80.43 ± 4.49 ng/L for EE2).

The relationship between types of hormones and their removal rate were examined by Kruskal–Wallis test. The results showed a significant correlation between types of hormones and their removal rate (p < 0.05). Figure 2 shows the removal percentage of hormones versus the type of hormone.

In review on steroid estrogens, researches reported that removal rate of E1, E2, E3 and EE2 in conventional WWTPs were not equal. Because type of hormone can affected on removal percentage of hormone [13,21,27,29].These results are in accordance with this study results.

Seasonal and temperature changes may affect the removal of estrogens from wastewater treatment plants. Usually, an increase in temperature leads to increase wastewater treatment efficiency as the metabolic rate of microorganisms in the various biological treatment plants increase. During winter, higher effluent concentrations for both natural and synthetic estrogens have been observed [30].

Shareef et al. [31] reported the concentration of E1 and E2 to be higher in winter compared with summer. They claim that it may be due to the dilution and transformation of some of these compounds during their transfer to WWTPs in the warm season [31]. The seasonal influent concentration of steroid estrogens E2, E3, and EE2 was reported by Zhou et al. [26] in the order of spring > autumn > summer > winter. However, Jin et al. [25] found a different result in a municipal sewage treatment plant in Wuhan, China, which indicated a lower influent concentration of E3 in summer than in winter. In this study, ANOVA test was used to examine the relationship between the two seasons (summer and autumn), and hormone concentrations. Results indicated that there is no significant correlation between hormone concentrations and seasons (p = 0.11). This may be due to small differences in temperature between these two seasons.

## Conclusions

Steroid hormones are a group of biologically active compounds that are excreted by human beings and animals. They enter the environment through sewage discharge and animal waste disposal and can affect human and wildlife health by disrupting their normal endocrine systems. In this research, the levels of E1, E2, E3 and EE2 in raw sewage influent and final treated effluent of 7 WWTPs across Tehran in two seasons (summer-autumn) were studied. The study showed that hormone concentrations in influents of these WWTPs were significantly different and WWTP number 7 had significant differences in influent hormone concentrations compared with others. This may be because it serves a community with bigger populations than other WWTPs. Results only showed a significant relationship between hormones and TSS removal rate, but there was no significant relationship between hormones and COD removal rate. Steroid hormone removal rates in WWTPs are dependent on the waste load and plant design. WWTP number 4 and 7 showed a significant removal rate for hormone, possibly because they have the highest removal rate for TSS than the others. Sorption on an organic-rich solid phase was found to be one of major mechanisms of estrogenic steroids removing in biological treatment and WWTP number 7 due to long sludge retention time (the relation between the method of wastewater treatment and the hormone concentration). The results showed a significant correlation between types of hormones and their respective removal rate. Studies on the relationship between seasons (summer and autumn) and hormone concentrations showed no significant correlation between hormone concentrations and seasons; also, the influent concentration and removal rates did not appear to be seasonally characteristic. As the effluent concentrations of steroid estrogens are high enough to cause adverse effects on the environment, current activated sludge processes should be optimised or other advanced treatment processes should be used to completely eliminate residual estrogens in WWTP effluents.

Consequently, urgent efforts are needed to determine cost-effective alternatives for the removal of these potentially harmful compounds from effluents and to establish corresponding regulations and instructions to control estrogen pollutants in Iran.

## Abbreviations

E1: Estrone; E2: 17β–estradiol; E3: Estriol; EE2: 17α–ethinylestradiol; EDCs: Endocrine disrupting compounds; WWTPs: Wastewater treatment plants; SPE: Solid-phase extraction; GC–MS: Gas chromatography–mass spectrometry; A2O: Anaerobic/anoxic/oxic; BOD5: Biochemical oxygen demand; COD: Chemical oxygen demand; TOC: Total organic carbon; TSS: Total suspended solids; BSTFA: N,O–bis(trimethylsilyl)trifluoroacetamide; TMCS: Trimethylchlorosilane; SIM: Selected ion monitoring.

## Competing interest

The authors declare that they have no competing interest.

## Authors’ contributions

The overall implementation of this study including design, experiments and data analysis, and manuscript preparation were the results of the corresponding author’s efforts. All authors have made extensive contribution into the review and finalization of this manuscript. All authors read and approved the final manuscript.
